# Giant Azygos Vein Aneurysm Mimicking a Mediastinal Mass With Segmental Partial Anomalous Pulmonary Venous Return

**DOI:** 10.7759/cureus.110859

**Published:** 2026-06-14

**Authors:** Akshay Makam, Gaurav Bhalla, Nikita Maria, Ahsan Mahmood, Muhammad Shahid

**Affiliations:** 1 Radiology, Russells Hall Hospital, Dudley, GBR; 2 Cardiology, Russells Hall Hospital, Dudley, GBR

**Keywords:** azygos vein aneurysm, chest radiography, incidental mediastinal mass, partial anomalous pulmonary venous return, thoracic venous anomalies

## Abstract

We present the case of an 87-year-old man in whom a giant azygos vein aneurysm was incidentally detected on chest radiography (CXR) performed following an acute stroke. The radiograph demonstrated a right paratracheal mediastinal opacity with attenuation similar to the adjacent aortic arch, raising suspicion of a vascular abnormality. Contrast-enhanced CT confirmed a large azygos vein aneurysm draining into the superior vena cava. Retrospective review demonstrated subtle right paratracheal prominence dating back to 2007, with gradual interval enlargement over more than a decade. No acquired cause was identified, and the patient remained asymptomatic throughout follow-up, supporting conservative management in selected patients. The patient also demonstrated concurrent isolated segmental partial anomalous pulmonary venous return (PAPVR) draining into the superior vena cava. This case highlights the importance of recognising azygos vein aneurysms as a differential for mediastinal masses on CXR and considering associated congenital vascular anomalies.

## Introduction

Azygos vein aneurysms are rare vascular anomalies that may mimic mediastinal masses on imaging [1,2]. They are often asymptomatic and discovered incidentally. The aetiology may be congenital or acquired, although the underlying mechanism remains unclear in many cases [1].

The majority of azygos vein aneurysms follow a benign course and are managed conservatively, though rare complications, including thrombosis and rupture, have been reported.

Accurate identification is important to differentiate these lesions from mediastinal neoplasms or lymphadenopathy and avoid unnecessary invasive investigations or surgical intervention [3,4]. We describe a case of an incidentally detected azygos vein aneurysm identified on chest radiography (CXR) and confirmed with contrast-enhanced CT, which was managed conservatively despite gradual interval enlargement over time.

## Case presentation

An 87-year-old man underwent a CXR in February 2019 to assess the nasogastric tube position following an acute stroke. His past medical history included cerebrovascular disease and longstanding wheeze.

Frontal CXR demonstrated an unexpected right paratracheal mediastinal opacity at the level of the aortic knuckle. The lesion demonstrated attenuation similar to the adjacent aortic arch and projected into the right upper thorax (Figure 1). Differential considerations included a vascular abnormality or mediastinal soft tissue mass.

**Figure 1:**
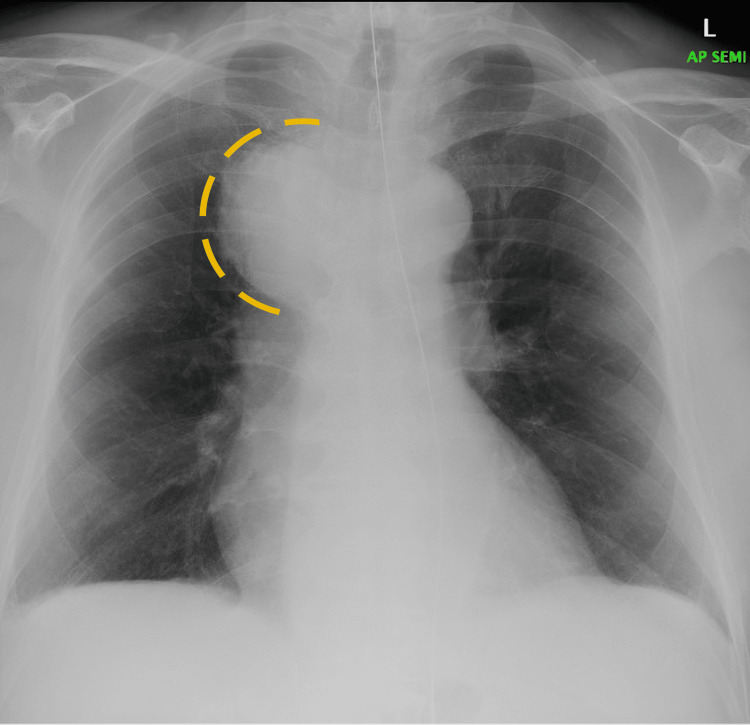
Chest radiograph from 2019 demonstrating a right paratracheal mediastinal opacity at the level of the azygos arch, indicated by dashed curved yellow lines.

Subsequent contrast-enhanced CT thorax demonstrated a large, well-defined, focally dilated azygos vein arch draining into the superior vena cava (SVC), with heterogeneous contrast mixing in keeping with sluggish venous flow, consistent with an azygos vein aneurysm (Figure 2). The aneurysm measured 9.5 cm × 3.7 cm. No intraluminal thrombus or other associated complications were identified.

**Figure 2:**
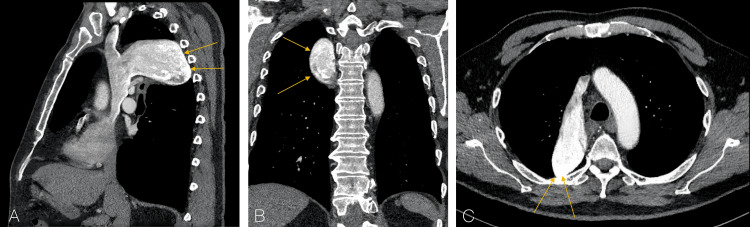
Contrast-enhanced CT thorax in sagittal (A), coronal (B), and axial (C) planes demonstrating a large, well-defined, focally dilated azygos vein arch consistent with an azygos vein aneurysm measuring 9.5 cm × 3.7 cm, indicated by yellow arrows. CT: computed tomography

A retrospective review of previous imaging demonstrated subtle right paratracheal prominence on chest radiographs dating back to 2009, consistent with a much smaller aneurysm that had not been appreciated at the time (Figure 3).

**Figure 3:**
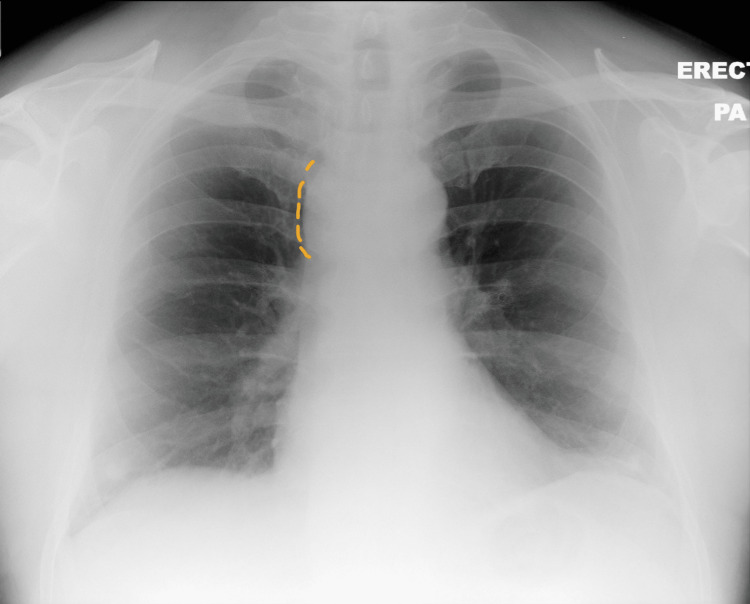
Chest radiograph from 2009. Dashed curved yellow lines demonstrating a mild right paratracheal bulge due to the azygos vein aneurysm, not appreciated at the time of reporting.

Given the longstanding incidental nature of the lesion and absence of symptoms or complications, conservative management was pursued. Serial imaging demonstrated gradual interval enlargement, with follow-up contrast-enhanced CT in July 2025, demonstrating a more saccular morphology measuring 9.7 cm × 5.8 cm (Figure 4).

**Figure 4:**
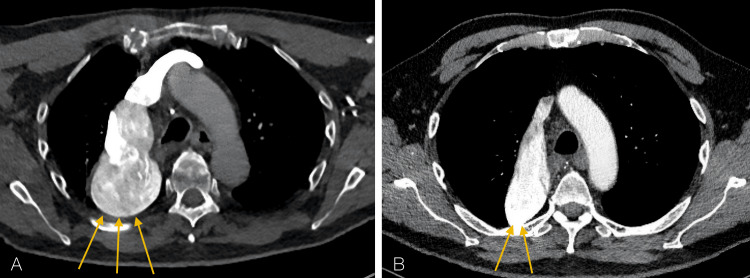
Contrast-enhanced CT thorax from 2025 (A) and 2019 (B) demonstrating interval enlargement and increasingly saccular morphology of the azygos vein aneurysm, indicated by yellow arrows. CT: computed tomography

The patient remained asymptomatic throughout follow-up. The diagnosis was made radiologically based on the characteristic enhancement pattern and continuity with the azygos venous system.

In addition, the patient demonstrated isolated anomalous venous drainage of the posterior segment of the right upper lobe into the SVC, consistent with segmental partial anomalous pulmonary venous return (PAPVR), which remained stable on serial imaging (Figure 5).

**Figure 5:**
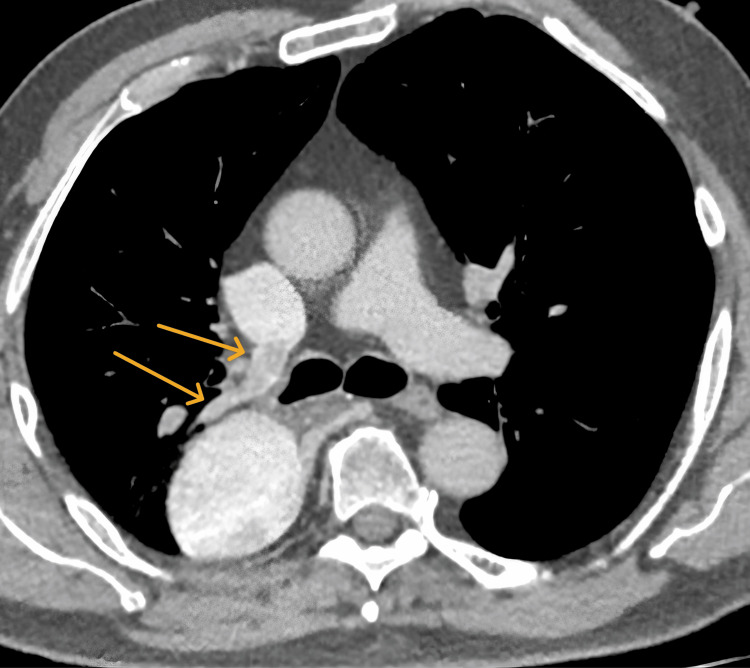
Contrast-enhanced CT in 2022 demonstrating isolated anomalous venous drainage of the posterior segment of the right upper lobe into the SVC, indicated by yellow arrows. CT: computed tomography; SVC: superior vena cava

## Discussion

A literature search of PubMed and Google Scholar was conducted using the terms 'azygos vein aneurysm', 'azygos varix', 'mediastinal mass mimic', and 'partial anomalous pulmonary venous return' to identify published cases and reviews relating to radiological mimics, aetiology, complications and management of azygos vein aneurysms.

Azygos vein aneurysms are rare anomalies of the thoracic venous system, which may be either congenital or acquired in origin. Most cases are acquired, typically resulting from pathological processes that elevate central venous pressure or impede venous return. These include congestive heart failure, portal hypertension, obstruction of the superior or inferior vena cava, and valvular heart disease [1]. Congenital aneurysms are less well-characterised but are thought to arise from focal weaknesses in the venous wall leading to aneurysmal dilatation, most commonly at the arch, as in our case [2,3]. The latter mechanism may account for the saccular morphology occasionally observed in these lesions.

Radiographically, azygos vein aneurysms typically present as right paratracheal or mediastinal soft-tissue masses, which may mimic more sinister entities such as lymphadenopathy or mediastinal neoplasms [2-4]. On plain chest radiographs, a helpful diagnostic clue is the lesion’s opacification, which mirrors that of adjacent vascular structures, raising suspicion for a vascular aetiology.

In several reported cases, surgical resection has been undertaken due to diagnostic uncertainty or concern regarding potential complications such as thrombosis or rupture [5,6]. Misdiagnoses have included bronchogenic cysts and pathological lymphadenopathy, often prompting unnecessary invasive interventions [7,8]. Where resection is required in symptomatic or complicated cases, a minimally invasive thoracoscopic (VATS) approach has been described as feasible, though care is warranted given the proximity of large mediastinal veins and the retroesophageal course of the azygos vein [5,6].

This case highlights the utility of high-resolution cross-sectional imaging in enabling a confident and non-invasive diagnosis. Contrast-enhanced CT confirmed an isolated azygos vein aneurysm with no identifiable acquired cause, suggesting an idiopathic or congenital aetiology in this elderly patient. Despite gradual interval enlargement over more than a decade, the patient remained asymptomatic and without associated complications, supporting conservative management. While rare complications, such as thrombosis or rupture, have been reported, the majority of azygos vein varices are asymptomatic and exhibit a stable natural history, rendering observation a reasonable management strategy in selected patients [1].

Although conservative management was deemed appropriate in this case, there are no formal guidelines regarding surveillance frequency; however, interval imaging would be warranted in the event of significant enlargement, new symptoms, thrombus formation, compression of adjacent structures or findings concerning for rupture.

Kurihara et al. described a similar case of an asymptomatic azygos vein aneurysm managed conservatively over a six-year period, with surgical resection ultimately performed following the incidental detection of thrombus formation on surveillance imaging [9]. This supports a strategy of active surveillance with a low threshold for intervention should complications arise.

The incidental finding of segmental anomalous pulmonary venous drainage was considered unrelated to the aneurysm, as it drained independently into the SVC without direct communication and was unlikely to significantly alter azygos venous haemodynamics.

Nevertheless, the concurrent finding of segmental PAPVR in this case may not be entirely coincidental. Although the azygos venous system and pulmonary veins arise from distinct embryological precursors - the cardinal and splanchnic venous systems, respectively - both undergo critical morphological development during the same gestational window, between the fourth and eighth weeks [10]. A shared susceptibility to dysregulation during this period of active vascular remodelling may provide a plausible, if speculative, basis for the co-existence of these two anomalies in a single patient [10,11].

While both anomalies have been reported independently in the literature, we are not aware of a previously reported case in which these two entities co-exist. This makes the present case unique and further highlights the importance of carefully assessing for associated congenital vascular anomalies once one abnormality has been identified.

## Conclusions

Azygos vein aneurysms are a rare but important differential for right-sided mediastinal masses on CXR. Attenuation similar to adjacent vascular structures should prompt consideration of a vascular origin, with contrast-enhanced CT essential for definitive diagnosis and characterisation. Conservative management is appropriate in asymptomatic patients, even in the setting of gradual interval enlargement, though continued surveillance is recommended with a low threshold for intervention should concerning clinical or radiological changes develop. This case also highlights the importance of assessing for associated congenital vascular anomalies, as demonstrated here by the concurrent finding of segmental PAPVR.
